# Neurological, Cognitive, and Psychological Findings Among Survivors of Ebola Virus Disease From the 1995 Ebola Outbreak in Kikwit, Democratic Republic of Congo: A Cross-sectional Study

**DOI:** 10.1093/cid/ciy677

**Published:** 2018-08-10

**Authors:** J Daniel Kelly, Nicole A Hoff, D’Andre Spencer, Kamy Musene, Matthew S Bramble, David McIlwain, Daniel Okitundu, Travis C Porco, George W Rutherford, M Maria Glymour, Zach Bjornson, Patrick Mukadi, Emile Okitolonda-Wemakoy, Garry P Nolan, Jean Jacques Muyembe-Tamfum, Anne W Rimoin

**Affiliations:** 1 School of Medicine, University of California, San Francisco; 2 School of Public Health, University of California, Los Angeles; 3 Department of Genetic Medicine Research, Children’s Research Institute, Children’s National Medical Center, Washington, D.C; 4 Department of Microbiology and Immunology, Stanford University, California; 5 Institut National de Recherche Biomédicale, Université de Kinshasa, Democratic Republic of Congo; 6 Faculté de Médecine, Université de Kinshasa, Democratic Republic of Congo; 7 Ecole de Sante Publique, Université de Kinshasa, Democratic Republic of Congo

**Keywords:** Ebola virus disease, survivors, Democratic Republic of Congo, clinical findings

## Abstract

**Background:**

Clinical sequelae of Ebola virus disease (EVD) have not been described more than 3 years postoutbreak. We examined survivors and close contacts from the 1995 Ebola outbreak in Kikwit, Democratic Republic of Congo (DRC), and determined prevalence of abnormal neurological, cognitive, and psychological findings and their association with EVD survivorship.

**Methods:**

From August to September 2017, we conducted a cross-sectional study in Kikwit, DRC. Over 2 decades after the EVD outbreak, we recruited EVD survivors and close contacts from the outbreak to undergo physical examination and culturally adapted versions of the Folstein mini-mental status exam (MMSE) and Goldberg anxiety and depression scale (GADS). We estimated the strength of relationships between EVD survivorship and health outcomes using linear regression models by comparing survivors versus close contacts, adjusting for age, sex, educational level, marital status, and healthcare worker status.

**Results:**

We enrolled 20 EVD survivors and 187 close contacts. Among the 20 EVD survivors, 4 (20%) reported at least 1 abnormal neurological symptom, and 3 (15%) had an abnormal neurological examination. Among the 187 close contacts, 14 (11%) reported at least 1 abnormal neurologic symptom, and 9 (5%) had an abnormal neurological examination. EVD survivors had lower mean MMSE and higher mean GADS scores as compared to close contacts (MMSE: adjusted coefficient: −1.85; 95% confidence interval [CI]: −3.63, −0.07; GADS: adjusted coefficient: 3.91; 95% CI: 1.76, 6.04).

**Conclusions:**

EVD survivors can have lower cognitive scores and more symptoms of depression and anxiety than close contacts more than 2 decades after Ebola virus outbreaks.

## Teaser

Ebola survivors can have lower cognitive scores and more symptoms of depression and anxiety than close contacts more than 2 decades after an outbreak, suggesting these areas as potential long-term needs for >10000 West African Ebola survivors.

Despite the more than 34 reported Ebola outbreaks since 1976 [1], we are only beginning to understand the long-term health consequences of survivorship of Ebola virus disease (EVD). A large proportion of EVD survivors experience ongoing, debilitating sequelae, which has been described in the short-term as a “post-EVD clinical syndrome” [2, 3]. Part of this clinical syndrome includes a spectrum of abnormal neurological, cognitive, and psychological findings, ranging from memory loss and meningoencephalitis to depressive symptoms, and these findings have been documented as early sequelae among EVD survivors [3–8]. A study conducted 29 months after the 2003 outbreak in Bundibugyo, Uganda, reported neurological and cognitive findings among EVD survivors [6], and a study conducted 1 month after the 1995 outbreak in Kikwit, Democratic Republic of Congo (DRC), reported psychological sequelae from EVD such as shame, anxiety, stigma, and rejection by family, friends, and neighbors [9], which have made reintegration challenging for survivors [10–12].

Older EVD outbreaks such as the 1995 outbreak in Kikwit may cause trauma and result in a significant burden of unrecognized, long-term mental health issues, such as depression and anxiety. In contrast to the West African outbreak of 2013–2016 [12, 13], international agencies did not provide clinical, psychosocial, or economic services to these older cohorts of EVD survivors to support them through their recovery and reintegration [14]. As a result, psychological consequences may be unresolved, whereas new psychological consequences may even occur from disability related to EVD clinical sequelae such as complications of uveitis [13, 15–17]. EVD-related neurological and cognitive sequelae might also occur within the same individuals, and post-traumatic syndromes have the potential to develop into chronic diseases.

The pathogenesis of neurological and cognitive sequelae is not well understood and, in rare circumstances, meningoencephalitis from viral persistence in the central nervous system (CNS) may have a role in early sequelae [8, 18]. Although neurological sequelae such as memory loss and confusion were reported more than 2 years after the 2003 Ugandan outbreak [6], it is unclear whether many neurological and cognitive sequelae are the result of CNS pathology or are manifestations of post-traumatic syndromes. This Ugandan study and the majority of others relied on self-reports rather than clinical examinations [5, 6]. Our understanding of the pathophysiological mechanisms of post-EVD sequelae is limited to uveitis and meningoencephalitis [19]. Thus, post-traumatic syndromes may play an underappreciated role in post-EVD syndrome and become complications leading to long-term neurological, cognitive, and psychological sequelae.

More than 2 decades after the 1995 Kikwit EVD outbreak, we conducted a study to describe the prevalence of adverse neurological, cognitive, and psychological findings and determine the association of EVD with these health outcomes.

## METHODS

### Ethics Statement

The study protocol was approved by the Institutional Review Boards at the Fielding School of Public Health at University of California, Los Angeles, in Los Angeles, California, USA, and the Kinshasa School of Public Health in Kinshasa, DRC. Written informed consent was obtained from all participants.

### Study Design and Participants

From August to September 2017, we conducted a cross-sectional study of EVD survivors and their close contacts from the 1995 EVD outbreak in Kikwit. During the outbreak, there were 315 EVD cases (89 laboratory-confirmed by presence of detectable antigen or immunoglobulin M [IgM] antibodies; 226 probable cases by World Health Organization [WHO] EVD case definition). The case fatality rate was 81%; thus, 61 cases survived EVD [20]. After the outbreak, these reported EVD survivors created the Kikwit Ebola Survivor Association. More than 2 decades later when we conducted our study, this organization provided us with a list of 42 EVD survivors who were reported EVD cases during the 1995 Ebola outbreak. Of these 42 members listed as part of the Kikwit Ebola Survivor Association, only 20 survivors were living in Kikwit and the surrounding area. We attempted to recruit these 20 survivors. Our social mobilization team announced recruitment through the Kikwit Ebola Survivor Association, media, community sensitization, and healthcare facilities in Kikwit and the surrounding area. We found that this list included survivors living in remote locations where there was no cell phone or radio coverage who were unable to be contacted. Thus, we enrolled an exhaustive, convenience sample from this list and defined them as reported EVD survivors.

During recruitment, there were a few individuals who self-identified as EVD survivors who reported direct contact with an EVD case and symptoms consistent with acute EVD during the post-exposure period; they met WHO EVD case definition [21] but were not on the list of “reported survivors” from the Kikwit Ebola Survivor Association. Unrecognized EVD cases and unknown sources of Ebola virus infection were reported during the 1995 Kikwit outbreak and have since been increasingly described as a public health issue of significance, particularly during the 2013–16 West Africa outbreak [22–25]. Based on our experiences in outbreak response work and unpublished findings from the 2013–16 West Africa outbreak, we rationalized that from the biosocial perspective, individuals who were perceived as EVD survivors in the community, regardless of being in a registry, were at risk for neurological, cognitive, and particularly psychological findings. We defined these individuals as self-reported EVD survivors. We included reported and self-reported EVD survivors in our study and defined them as EVD survivors.

Close contacts were included in the study if they reported direct contact with an EVD case and reported that this contact event occurred during the 1995 EVD outbreak in Kikwit. Direct contact was defined as directly touching body fluids (including but not limited to blood, saliva, mucus, vomit, urine, or feces) from an infected person (alive or dead) and having touched someone’s eyes, nose, or mouth or an open cut, wound, or abrasion [26]. Recruitment of close contacts primarily occurred using 2 different methods; EVD survivors identified up to five individuals who were in contact with them during their acute illness, and in collaboration with the leadership of Kikwit General Hospital, we obtained a list of healthcare workers who worked in Ebola care facilities during the 1995 outbreak in Kikwit and the surrounding vicinity. For individuals identified by EVD survivors, contact mostly occurred in the community and particularly in households of EVD survivors. For healthcare workers, contact mostly occurred in healthcare settings.

Each participant responded to a questionnaire and received a physical examination. The sociodemographic and epidemiological questionnaire was administered by study staff who were not healthcare providers. We recruited and trained Congolese doctors, who were medical officers, to perform the medical history and physical examination and to administer the cognitive and psychological questionnaires. Doctors with specialty training in Ebola clinical sequelae were involved in the training and supervision of data collection.

### Data Collection

The outcome measurements included neurological, cognitive, and psychological findings. We used self-reported symptoms and physical examinations to assess for abnormal neurological findings. Abnormal neurological symptoms were defined as the self-reported symptoms that were endorsed from a 12-item checklist. The list of symptoms included headache, syncope, dizziness, vertigo, seizures, diplopia, paresthesia, paralysis, weakness in any limbs, tremor, ataxia, and memory loss. Abnormal neurological examinations were defined as physical exam findings of the neurologic system that were assessed from an 8-item checklist. The exam assessed speech, tremor, reflexes, cranial nerves, focal weakness, gait/balance, sensory, and other abnormal findings (free response).

Cognitive findings were defined by the Folstein mini-mental status exam (MMSE) [27]. Cronbach’s alpha for the MMSE was 0.58. We used Goldberg anxiety and depression scale (GADS), which was developed from 36 items in the Psychiatric Assessment Schedule [28] and has been used in several community-based studies of older adults [29]. This assessment tool was validated in a 2012 study in Kinshasa, DRC [30]. GAD was an 18-item scale that consisted of a 9-item depressive symptom subscale and 9-item anxious symptoms subscale. Cronbach’s alpha for GADS was 0.86, whereas the depressive symptom subscale and anxious symptoms subscale were 0.86 and 0.84, respectively. EVD-related stigma was defined based on a 7-item index adapted from the People Living with HIV/AIDS Stigma for EVD survivors of the West Africa outbreak [31]. Cronbach’s alpha for the stigma scale was 0.76. Confounders were identified from the questionnaire and included current age, sex, educational level, marital status, and prior healthcare worker status (during the outbreak).

### Statistical Analyses

We described the characteristics and neurological, cognitive, and psychological findings of participants. Age, MMSE (scale, 0–30), GADS (scale, 0–18), GADS subscales (each scale, 0–9) and the 7-item EVD-related stigma index (scale, 0–7) were continuous variables. Sex, educational level, marital status, and type of occupation were dichotomized. Characteristics among EVD survivors and close contacts were assessed for differences using Fischer exact or *t*-tests. We assessed the association between EVD survivorship (EVD survivors vs close contacts) and health outcomes and used linear regression models in bivariate and multivariate analyses adjusting for the potential confounders of age, sex, educational level, marital status, and being a healthcare worker. Our threshold for a statistically significant finding was *P* < .05. We conducted these analyses with EVD survivors (self-reported and reported), as well as reported EVD survivors. We performed sensitivity analyses for EVD survivors, using a propensity score model (Supplementary Material 1 and 2) and a sensitivity analysis for close contacts by comparing characteristics of individuals identified by EVD survivors versus list of healthcare workers (Supplementary Material 3). Analyses were performed using STATA/IC 13.1 (STATA Corporation, College Station, TX) and R (version 3.2.3).

## RESULTS

Twenty EVD survivors and 187 close contacts were enrolled. Among these 20 EVD survivors, the mean age was 53.2 (95% confidence interval [CI], 48.1–58.3). The majority (70%) was female, whereas nearly half (45%) had completed only primary school or had no formal education. Nine (45%) were married, and 8 (40%) were healthcare workers during the outbreak. Comparing these characteristics among EVD survivors and close contacts, there was a trend of a difference in educational level (Table 1). In sum, 14 (70%) of the 20 EVD survivors were reported cases. There was no difference in characteristics among these groups (Table 1).

**Table 1.** Characteristics of Ebola Virus Disease (EVD) Survivors (N = 20), Reported EVD Survivors (N = 14) and Close Contacts (N = 187) and Prevalence of Neurological, Cognitive, and Psychological Findings

Characteristics	EVD Survivors		Reported EVD Survivors		Close Contacts		*P* Values for Survivors	*P* Values for Reported Survivors
	N = 20	% or 95% CI	N = 14	% or 95% CI	N = 187	% or 95% CI		
Current age, years (mean)	53.2	48.1–58.3	53.4	46.1–60.6	53.5	51.6–55.3	.94	.98
Female	14	70.0	12	85.7	99	52.9	.16	**.02**
Education: none or primary	9	45.0	8	57.1	44	23.5	.06	**.01**
Married at present	9	50.0	6	42.9	117	62.6	.23	.16
Healthcare worker in 1995	8	44.4	5	35.7	83	44.4	.82	.59
Abnormal neurological symptoms	4	20.0	3	21.4	14	10.9		
Abnormal neurological examination	3	15.0	2	14.0	9	4.8		
MMSE^a^ (mean)	20.6	17.6–23.6	20.1	16.4–23.7	23.5	22.8–24.1		
GADS^b^ (mean)	8.0	5.4–10.6	9.8	7.1–12.5	4.1	3.3–4.9		
GADS: depressive symptoms sub-scale (mean)	3.4	2.1–4.6	4.2	2.7–5.8	1.6	1.2–2.0		
GADS: anxious symptoms sub-scale (mean)	4.7	3.2–6.1	5.5	4.2–7.0	2.5	2.0–2.9		
Any EVD-related stigma in the 6 months after the outbreak?	13	65.0	9	64.3				
Any EVD-related stigma in the past 6 months?	1	5.0	0	0				

Bold indicates a *P* value <.05.

Abbreviations: CI, confidence interval; EVD, Ebola virus disease; GADS, Goldberg anxiety and depression scale; MMSE, mini-mental status exam.

^a^Folstein MMSE; scale 0–30.

^b^GADS; scale 0–18.

### Description of Neurological, Cognitive, and Psychological Findings

Among EVD survivors, 4 (20%) reported at least 1 abnormal neurological symptom, which included headache (15%), vertigo (15%), paresthesia (5%), weakness in any limbs (5%), tremor (5%), and ataxia (5%). Three (15%) survivors had an abnormal neurological examination, which included the following findings among survivors: tremor (10%), gait/balance disturbance (10%), speech abnormality (5%), cranial nerve abnormality (5%), focal weakness (5%), and sensory deficit (5%). Mean MMSE score was 20.6 (95% CI, 17.1–23.6), and mean GADS score was 8.0 (95% CI, 5.4–10.6). Mean GADS subscale score for depressive symptoms was 3.4 (95% CI, 2.1–4.6) and mean GADS subscale score for anxious symptoms was 4.7 (95% CI, 3.2–6.1). Thirteen (65%) reported experiencing at least 1 item of EVD-related stigma in the 6 months after outbreak, and one individual experienced EVD-related stigma in the 6 months prior to the interview (Table 1).

Among close contacts, 14 (11%) reported at least 1 abnormal neurologic symptom, which included headache (10%), vertigo (4%), paresthesia (2%), weakness in any limbs (2%), ataxia (2%), and memory loss (2%). Nine (5%) had an abnormal neurological examination, which included the following findings among contacts: abnormal reflexes (4%), cranial nerve abnormality (4%), gait/balance disturbance (3%), tremor (2%), sensory deficit (2%), and focal weakness (1%). Mean MMSE score was 23.5 (95% CI, 22.8–24.1). Mean GADS score was 4.1 (95% CI, 3.3–4.9), and mean GADS subscale score for depressive symptoms was 1.6 (95% CI, 1.2–2.0) and mean GADS subscale score for anxious symptoms was 2.5 (95% CI, 2.0–2.9) (Table 1).

### Associations With Neurological, Cognitive, and Psychological Findings

In unadjusted analyses, EVD survivors had a statistically lower mean MMSE score, higher mean GADS score, and higher mean GADS subscale score as compared to close contacts. After multivariable adjustment, EVD survivors were at risk of a lower mean MMSE score as compared to close contacts (adjusted coefficient: −1.85; 95% CI: −3.63, −0.07). EVD survivors were at risk of a higher mean GADS score as compared to close contacts (adjusted coefficient: 3.91; 95% CI: 1.76, 6.04). EVD survivors also had higher mean GAD subscale scores for depressive symptoms (adjusted coefficient: 1.80; 95% CI: 0.70, 2.90) and for anxious symptoms (adjusted coefficient: 1.98; 95% CI: 0.75, 3.21) than close contacts. A similar pattern of associations, some of which had stronger magnitudes, was also observed when comparing reported EVD survivors to close contacts. There were no significant associations between survivorship and abnormal neurological symptoms or abnormal neurological examinations (Table 2).

**Table 2.** Association of Ebola Virus Disease (EVD) Survivorship With Neurological, Cognitive, and Psychological Findings (EVD Survivors, N = 20; Reported EVD Survivors, N = 14; Close Contacts, N = 187).

Outcomes	Adjusted Coefficient for EVD Survivors (95% CI)	*P* Values	Adjusted Coefficient for Reported EVD Survivors (95% CI)	*P* Values
Abnormal neurologic symptoms	0.62 (−0.67–1.92)	.35	0.51 (−0.99–2.00)	.51
Abnormal neurological examination	1.57 (−0.21–3.35)	.08	1.11 (−1.10–3.33)	.32
MMSE^a^	−1.85 (−3.63–-0.07)	**.04**	−2.10 (−4.05–-0.14)	**.04**
GADS ^b^	3.91 (1.76–6.04)	**<.01**	5.89 (3.41–8.37)	**<.01**
GADS: Depressive symptoms subscale	1.80 (0.70–2.90)	**<.01**	2.77 (1.47–4.07)	**<.01**
GADS: Anxious symptoms subscale	1.98 (0.75–3.21)	**<.01**	3.04 (1.61–4.47)	**<.01**

Covariates used to adjust for confounding included age, sex, educational level, marital status, and being a healthcare worker. *P* values are described for adjusted models, and bold indicates a value <.05.

Abbreviations: CI, confidence interval; EVD, Ebola virus disease; GADS, Goldberg anxiety and depression scale; MMSE, mini-mental status exam.

^a^Folstein MMSE, scale 0–30.

^b^GADS, scale 0–18.

## DISCUSSION

This is the first follow-up study of EVD survivors and close contacts from the 1995 Kikwit outbreak since 1997 [5]. Although adverse neurological, cognitive, and psychological findings have been described shortly after surviving EVD, we found EVD survivors to have reduced general cognition and more symptoms of depression and anxiety than close contacts more than 2 decades after the Kikwit outbreak. Based on our small sample, there was no evidence of significantly elevated risk for other abnormal neurological findings or persistent EVD-related stigma. These findings were consistent between self-reported and reported EVD survivors. Given the small size of the EVD survivor cohort from Kikwit, similar studies with larger sample sizes will be beneficial to expand these findings.

More than 2 decades after the outbreak, our findings suggest that EVD survivors continue to be in need of psychosocial support and mental health intervention. The majority of reported and self-reported EVD survivors in our study recalled experiences of EVD-related stigma and discrimination. Even if not still ongoing, community perceptions of survivorship as well as outbreak experiences may have contributed to cognitive and psychological findings. In our conversations with these survivors, many experienced post-traumatic syndromes during the outbreak and post-outbreak period without receiving adequate care. Other than the West Africa outbreak, international agencies have not provided free services to EVD survivors. There may be large, unaddressed needs in this field. Moreover, these psychosocial needs may be ongoing for long periods of time for some EVD survivors. Studies assessing the neurological, cognitive, and psychological health of EVD survivors across all prior outbreaks are needed.

We conducted analyses with EVD survivors and their subgroup of reported EVD survivors as well as with propensity score models and the consistency of findings across outcomes and subgroups was reassuring that our findings are not due to type I or II errors. Due to the very small numbers of self-reported EVD survivors, we were unable to calculate effect estimates for this group without including reported EVD survivors. Given that the study was cross-sectional, we are unable to determine when the outcomes occurred or how long they persisted. No data were available for comparison related to the pre-outbreak, outbreak or early post-outbreak periods. We used GADS because it was adapted to the DRC; GADS is a measure of symptoms and not a DSM-5 diagnosis. Our measurement tools (scales and index) had high Cronbach’s alpha scores, indicating an acceptable level of internal consistency. These tools have been validated in our settings including sub-Saharan Africa but were not validated specifically for this study population. There may have also be recall error for some of the measurements such as having ever experienced stigma. Attempts were made to limit recall error by designing questions to report responses that were current at the study visit, particularly for clinical questions. Furthermore, our findings represent a small population of older EVD survivors and cannot necessarily be generalized to other EVD survivor populations. The use of close contacts as a control group served as an important strength in our methodology to determine the predictors of EVD survivorship amongst neurological, cognitive, and psychological data.

This study highlights general cognition and symptoms of depression and anxiety as areas where there may be long-term health needs of >10000 EVD survivors from the 2013–16 West Africa outbreak and for the EVD survivors of >33 other EVD outbreaks. More studies are needed to understand other long-term health outcomes of EVD survivors, but our findings are a call to action for the scientific community, as well as international and government agencies to work in collaboration to understand and address the long-term needs of EVD survivors no matter the size of the outbreak.

## Supplementary Data

Supplementary materials are available at *Clinical Infectious Diseases* online. Consisting of data provided by the authors to benefit the reader, the posted materials are not copyedited and are the sole responsibility of the authors, so questions or comments should be addressed to the corresponding author.

Supplementry Material

## Notes

***Acknowledgments.*** We appreciate the Ebola virus disease survivors and close contacts who participated in the study. In addition, we thank Drs Desire Tshala, Dieudonnne Mumba, Catherine Oldenburg, Alison Huang, Raj Kalapatapu, Susan Kegeles, and Willi McFarland, as well as Faucett Catalyst Fund and the entire University of California, Los Angeles-Democratic Republic of the Congo research program and staff for their support and input.

***Financial support.*** M. S. B. is supported by the Fogarty International Center of the National Institutes of Health (NIH) under award number D43TW009343 and the University of California Global Health Institute (UCGHI); Faucett Catalyst Fund, University of California, Los Angeles, and United States Food and Drug Administration (HHSF223201610018C) are funding sources.

***Potential conflicts of interest.*** None declared. All authors have submitted the ICMJE Form for Disclosure of Potential Conflicts of Interest. Conflicts that the editors consider relevant to the content of the manuscript have been disclosed.
