# Supplementary material for: Neurological, Cognitive, and Psychological Findings Among Survivors of Ebola Virus Disease From the 1995 Ebola Outbreak in Kikwit, Democratic Republic of Congo: A Cross-sectional Study
Source: Clin Infect Dis. 2018 Aug 10;68(8):1388–93. doi: 10.1093/cid/ciy677 (PMC6452000; doi:10.1093/cid/ciy677)
Supplement: Supplementry Material [file ciy677_suppl_neuropsych_supplement_clean.docx]

**Supplement.**

**Supplement 1.** Association of EVD survivorship with neurological, cognitive, and psychological findings using propensity score models (EVD survivors, N=20; close contacts, N=187).

| **Outcomes** | **Unadjusted Coefficient (95% CI)** | **Adjusted Coefficient (95% CI)** | **p-values** |
| --- | --- | --- | --- |
| Abnormal neurologic symptoms | 0.71 (-0.52, 1.94) | 0.71 (-0.58, 1.99) | 0.28 |
| Abnormal neurological examination | 1.25 (-0.15, 2.65) | 0.81 (-0.87, 2.48) | 0.35 |
| MMSE^1^ | -2.86 (-4.88, -0.84) | -2.05 (-4.00, -0.10) | **0.04** |
| GADS^2^ | 3.90 (1.73, 6.06) | 4.46 (2.17, 6.76) | **<0.01** |
| GADS: Depressive symptoms sub-scale | 1.74 (0.62, 2.85) | 2.00 (0.81, 3.20) | **<0.01** |
| GADS: Anxious symptoms sub-scale | 2.20 (0.97, 3.42) | 2.47 (1.18, 3.76) | **<0.01** |

^1^Folstein mini-mental status exam (MMSE; scale 0-30)

^2^Goldberg Anxiety and Depression Scale (GADS; scale 0-18).

**Supplement 2.** Association of reported EVD survivorship with neurological, cognitive, and psychological findings using propensity score models (reported EVD survivors, N=14; close contacts, N=187).

| **Outcomes** | **Unadjusted Coefficient (95% CI)** | **Adjusted Coefficient (95% CI)** | **p-values** |
| --- | --- | --- | --- |
| Abnormal neurologic symptoms | 0.80 (-0.59, 2.19) | 0.55 (-0.91, 2.00) | 0.46 |
| Abnormal neurological examination | 1.19 (-0.45, 2.83) | 1.10 (-0.63, 2.82) | 0.21 |
| MMSE^1^ | -3.38 (-5.59, -1.17) | -2.33 (-4.49, -0.16) | **0.04** |
| GADS^2^ | 5.68 (3.23, 8.13) | 5.55 (3.02, 8.09) | **<0.01** |
| GADS: Depressive symptoms sub-scale | 2.60 (1.31, 3.89) | 2.62 (1.29, 3.96) | **<0.01** |
| GADS: Anxious symptoms sub-scale | 3.12 (1.74, 4.50) | 2.94 (1.52, 4.36) | **<0.01** |

^1^Folstein mini-mental status exam (MMSE; scale 0-30)

^2^Goldberg Anxiety and Depression Scale (GADS; scale 0-18).

**Supplement 3.** Characteristics of close contacts identified by EVD survivors (N=76) and identified by a list of healthcare workers (N=111) and prevalence of neurological, cognitive, and psychological findings. Bold indicates a p-value<0.05.

|  | **Close contacts identified by EVD survivors** | | **Close contacts identified by list of healthcare workers** | | **p-values** |
| --- | --- | --- | --- | --- | --- |
| **Characteristics** | **N=76** | **% or** 95% **CI** | **N=111** | **% or** 95% **CI** |  |
| Current age, years (mean) | 54.0 | 50.9-57.2 | 53.1 | 50.7-55.4 | 0.62 |
| Female | 39 | 51.3 | 60 | 54.1 | 0.77 |
| Education: None or Primary | 25 | 32.9 | 19 | 17.1 | **0.02** |
| Married at present | 51 | 67.1 | 66 | 59.5 | 0.36 |
| Abnormal neurological symptoms | 7 | 9.2 | 7 | 6.3 | 0.62 |
| Abnormal neurological examination | 3 | 4.0 | 6 | 5.4 | 0.74 |
| MMSE^1^ (mean) | 23.4 | 22.6-24.2 | 23.5 | 22.5-24.5 | 0.85 |
| GADS^2^ (mean) | 4.4 | 3.4-5.5 | 3.8 | 2.7-5.0 | 0.45 |
| GADS: depressive symptoms sub-scale (mean) | 1.5 | 1.0-2.1 | 1.7 | 1.1-2.3 | 0.75 |
| GADS: anxious symptoms sub-scale (mean) | 2.8 | 2.2-3.4 | 2.1 | 1.5-2.8 | 0.12 |

^1^Folstein mini-mental status exam (MMSE; scale 0-30)

^2^Goldberg Anxiety and Depression Scale (GADS; scale 0-18).
